# *Mycobacterium abscessus* research: learning from challenges

**DOI:** 10.1128/jb.00436-25

**Published:** 2026-02-04

**Authors:** Ryan Z. Treen, Mercedes Gonzalez-Juarrero, Mary Jackson, Pascal Lapierre, Laurent Kremer, Pallavi Ghosh, Anil K. Ojha

**Affiliations:** 1 Wadsworth Center, New York State Department of Health 116287, Albany, New York, USA; 2 Mycobacteria Research Laboratories, Department of Microbiology, Immunology and Pathology, Colorado State University 3447, Fort Collins, Colorado, USA; 3 Centre National de la Recherche Scientifique UMR 9004, Institut de Recherche en Infectiologie de Montpellier (IRIM), Université de Montpellier, Montpellier, France; 4 INSERM, IRIM, Montpellier, France; 5 Department of Biomedical Sciences, University at Albany 1084, Albany, New York, USA; University of Notre Dame, Notre Dame, Indiana, USA

**Keywords:** Non-tuberculous mycobacteria, *Mycobacterium abscessus*

## Abstract

*Mycobacterium abscessus (Mab),* a rapidly growing mycobacterial species with intrinsic and acquired resistance to multiple antibiotics, is an emerging public health concern. The rise in clinical cases of treatment-refractory infections of *M. abscessus* has propelled its research toward novel therapeutic approaches. The number of publications entitled “*Mycobacterium abscessus”* has increased by ~300% over the last decade, of which the majority of studies exploring the fundamental biology and pathogenesis of Mab have used the reference strain ATCC19977. However, whole-genome sequence analyses, combined with transposon-seq based functional genomics, reveal an open pan-genome with significant variations in the essential genes across ATCC19977 and clinical isolates. These new discoveries demand a careful selection of strains and growth conditions in experimental design. In this minireview, we discuss these challenges and propose a framework for future *M. abscessus* studies *in silico*, including a new web-based resource for pangenome analysis, *in vitro,* and in animal models.

## INTRODUCTION

The genus Mycobacterium represents over 200 species, most of which innocuously inhabit our environment, while some like *Mycobacterium tuberculosis* and *Mycobacterium leprae* have killed or stigmatized humankind for ages (1, 2). However, recent decades have witnessed a significant surge in infections caused by non-tuberculous mycobacteria (NTMs) in people with underlying health conditions like cystic fibrosis (CF) or chronic obstructive pulmonary disease (COPD) (3–10). Infection with *Mycobacterium avium* and *Mycobacterium abscessus (Mab)* represents over 75% of all NTM infections in humans (11), of which *Mycobacterium abscessus* is emerging as a serious public health concern due to its total recalcitrance to drugs used for the treatment of *M. tuberculosis* infection (12). *M. abscessus* belongs to Runyon group IV complex of rapidly growing mycobacteria found predominantly in soil and water environment, with an ability to also cause chronic skin and soft tissue infections in humans (13–15). The current treatment regimen for *M. abscessus* infections entails a combination of an oral macrolide in conjunction with the aminoglycoside, amikacin, and one or more of the injectables—cefoxitin, imipenem, and tigecycline—for a period of 6–12 months (5, 9, 16). Despite such aggressive treatments, the average rate of eradication is mere ~45% (5, 17, 18), highlighting the urgent need for effective therapeutics against *M. abscessus*, for which it becomes necessary to gain molecular insights into its physiology, drug resistance mechanisms, and pathogenesis.

Crucial insights were obtained from the genome sequence of the type strain of *M. abscessus*, ATCC19977 (also called CIP104536^T^ in the collection of the Pasteur Institute) (19). The genetic similarity between *M. abscessus* and other mycobacterial species, particularly the rapidly growing and genetically tractable model species *Mycobacterium smegmatis* used for fundamental biological studies of mycobacteria (20), has allowed the genetic tools developed for *M. smegmatis* to kick-start genetic studies of *M. abscessus* involving gene replacement (21), gene expression (22), gene-expression interference (23, 24), and genome-wide transposon mutagenesis (25–27). These genetic approaches have led to discoveries of gene function in *M. abscessus* physiology and antibiotic resistance and have developed a new understanding of the mechanisms of action of new and established drugs. Equally important insights into the *M. abscessus* biology and evolution can be obtained from the differences between the *M. smegmatis* mc^2^155 and *M. abscessus* ATCC19977 genomes (19). For example, homologs of some of the virulence-associated genes like *virS* and *sigC* of *M. tuberculosis* are found in *M. abscessus,* but not in *M. smegmatis* (19), suggesting that the presence of these genes could be important for the survival of *M. abscessus* in the host. Moreover, the presence of 17 gene clusters in the ATCC19977 genome that are syntenic to the genomic regions of non-mycobacterial species, including *Pseudomonas aeruginosa,* offers the evidence of horizontal gene transfer (HGT) in *M. abscessus* in the environment (19).

The genome sequence of the type strain ATCC19977, combined with the rapid evolution of cost-effective high-throughput sequencing technologies, ushered in a new phase of population genomics studies of *M. abscessus* that involved multi-tiered whole-genome sequence (WGS) analyses of over thousand clinical *M. abscessus* isolates from both single and multiple geographical locations (28–32). These studies reveal a highly diverse genomic architecture across individual isolates that are marked by saltatory changes likely arising from HGT. Moreover, pan-genome analyses reveal only ~70% of the genome to be conserved in all isolates, thus called a “core genome” and remaining as an “accessory genome” (33). Furthermore, a recent high-throughput transposon-insertion sequencing of 25 clinical isolates reveals a distinct set of essential genes for individual isolates (26), suggesting independent evolution of these isolates from their environmental ancestors living in distinct niches. These findings broadly highlight the pitfalls of using the type strain as a model system for studies on pathogenesis and antibiotic resistance in *M. abscessus*. In this minireview, we discuss these challenges while proposing new considerations for future *M. abscessus* studies.

## DISCOVERY AND CLASSIFICATION OF *MYCOBACTERIUM ABSCESSUS*

*Mycobacterium abscessus* was first recognized as a unique mycobacterial species in 1953 by Moore and Frerichs, who isolated it from a knee abscess of a 63-year-old woman (34). The authors determined that, despite histological similarity with *M. tuberculosis,* these bacilli exhibited rapid growth and therefore required an entirely new classification. In their justification for a new name for the isolate, Moore and Frerichs also challenged the prior classification method of acid-fast bacilli (35), which by then had already classified many mycobacterial species including *Mycobacterium thamnopheos*, *Mycobacterium chelonei*, and *Mycobacterium ranae*, which was later reclassified as *Mycobacterium fortuitum* (36). Subsequent taxonomic analysis showed strong similarity between *M. chelonei* and *M. abscessus*, which resulted in the demotion of *M. abscessus* to a subspecies of the overarching taxon *M. chelonei*, of which *M. chelonei* subsp. *chelonei* was also included (13). This study relied on qualitative data such as pigment, enzymatic activity, growth on a panel of carbon and/or nitrogen sources, and acid production from a panel of sugars (13). A genetic analysis of *M. chelonae* subsp. *chelonae* and *M. chelonae* subsp. *abscessus,* performed two decades later, found only 35% DNA relatedness between these two, thereby re-establishing *M. abscessus* as a distinct species (37). In 2004, Drancourt and colleagues discovered an unusual mycobacterial isolate with 96% and 98% similarity to *M. abscessus rpoB* and *recA* genes, respectively, thereby naming it *Mycobacterium massiliense* (38). The same group performed another genetic analysis of 59 rapidly growing mycobacterial species and found another novel isolate with 95.6% sequence similarity with the *M. abscessus rpoB* gene, designating it as *Mycobacterium bolletii* (39). However, a WGS of 168 isolates of *M. abscessus* and subsequent phylogenetic analysis showed three distinct clusters of *M. abscessus abscessus*, *M. abscessus massiliense,* and *M. abscessus bolletii,* while all three had over 99% genome-wide similarity (30)*,* although individual strains from each of these three subspecies show extensive genome-wide mosaicism indicating HGT (40). Based on these data and a previous proposal (41)*, M. abscessus bolletii* and *M. abscessus massiliense* are now two distinct subspecies of *M. abscessus,* with *M. abscessus abscessus* itself being the third subspecies. Importantly, unlike most *M. abscessus* subsp. *abscessus* and *M. abscessus* subsp. *bolletii* strains*, M. abscessus* subsp. *massiliense* carries a loss-of-function mutation in the *erm41* gene, which is responsible for conferring inducible macrolide resistance through methylation of the 23S ribosomal RNA (42).

We note that Gupta et al. have recently proposed a new classification scheme that splits *Mycobacterium* into five genera: *Mycolicibacter, Mycolicibacterium, Mycolicibacillus, Mycobacteroides*, and *Mycobacterium* (43, 44), leading to subsequent incorporation of these nomenclatures in major databases such as the National Center for Biotechnology Information and causing significant confusion among the scientific community. However, a consortium specializing in mycobacterial taxonomy chose to ignore the new classification scheme as it was causing inconsistencies in communication and maintenance of the clinical database (45). Therefore, we too have chosen to retain *Mycobacterium* over the nomenclature proposed by Gupta et al. (43, 44).

## CLINICALLY RELEVANT KEY FEATURES OF *M. ABSCESSUS*

*M. abscessus* infections in humans manifest several treatment challenges that likely originate from the intrinsic and acquired phenotypes of the pathogen as discussed below.

### Extreme antibiotic resistance

The innate antibiotic resistance of *M. abscessus*, not typically observed in other pathogenic mycobacterial species, has been attributed to an impermeable cell envelope as well as to a combination of chromosomally encoded efflux systems, as well as enzymes that modify either the drug or the target and are expressed either constitutively or induced by the antibiotic exposure itself. Additionally, *M. abscessus* also displays genetic polymorphisms of target genes, prominently within the ethambutol and quinolone resistance-determining region. Specifically, I303Q and L304M of arabinosyl transferase, A83 of *gyrA,* and R447 and N464 of *gyrB* result in high-level resistance to ethambutol and fluoroquinolones, respectively (46, 47). Mechanisms of antibiotic resistance in *M. abscessus* can be broadly classified into the following four categories.

#### Antibiotic modification

*M. abscessus* encodes several enzymes that can modify and inactivate different classes of antibiotics, most prominently the aminoglycosides, rifamycins, tetracyclines, and β-lactams, rendering these antibiotics unavailable for use in therapy. Aminoglycosides, a large group of drugs targeting the 16S rRNA of the 30S ribosomal subunit, are modified at their hydroxyl and amino groups by phosphotransferases and acetyltransferases. MAB_4395 encodes a 2′-*N*-acetyltransferase that acetylates gentamycin, tobramycin, and kanamycin (48), MAB_4532c (encoded by *eis2*) modifies kanamycin, hygromycin, and amikacin (48), and the 3″ *O*-phosphotransferase encoded by *MAB_2385* modifies streptomycin (49). Deletion of these genes results in increased susceptibility of *M. abscessus* to the respective antibiotics. Rifamycins, that target the β subunit of RNA polymerase, while being the frontline drug against *M. tuberculosis*, are ineffective against *M. abscessus*, primarily due to ADP-ribosylation of these drugs by MAB_0591. Deletion of *MAB_0591* greatly increases the susceptibility of *M. abscessus* to rifampicin, rifabutin, and rifapentine (50–52). Homologs of *MAB_0591*, while present in several rapidly growing NTMs, are absent in other pathogenic mycobacteria such as *M. avium, M. ulceran*s, and *M. tuberculosis*. Tigecycline, a glycylcycline tetracycline, is effective against *M. abscessus* infections. However, previous generations such as doxycycline and tetracycline are rendered ineffective due to the presence of *MAB_1496c* (*MabTetX*), which monooxygenates these drugs and triggers their degradation. Deletion of *MAB_1496c* results in a greatly enhanced sensitivity to these tetracyclines (53). *M. chelone*, *M. fortuitum,* and *M. abscessus* are the only mycobacterial species that appear to be encoding *MabTetX*, although *MabTetX* homologs are curiously present in other actinobacteria and a CF pathogen *Burkholderia spp* (53). The majority of β-lactam antibiotics, which target peptidoglycan synthesis in bacteria, are ineffective against *M. abscessus*. This is attributed to the constitutive expression of the Ambler class A β-lactamase (*MAB_2875*/Bla_Mab_), which confers resistance to a broad spectrum of β-lactams and closely resembles the β-lactamases from *Pseudomonas* and *Serratia* spp. Cefoxitin and imipenem, while also substrates of Bla_Mab_, are hydrolyzed at a slow rate and show a moderate effect *in vitro* (54). A study demonstrating the effectiveness of avibactam, a β-lactamase inhibitor, provides hope in repurposing the β lactams or increasing the effectiveness of cefoxitin and imipenem (55).

#### Target modification

Macrolides are the most common antibiotic in the multidrug regimen prescribed against *M. abscessus* infections. However, their use is restricted by the presence of several innate mechanisms that reduce the efficacy of the drug. Most prominently, a ribosomal methyltransferase (encoded by *erm41*) that methylates the A2058 nucleotide of the 23S rRNA confers simultaneous resistance to macrolide-lincosamide-streptogramin-ketolide-oxazolidinone groups of antibiotics that have overlapping binding sites with A2058 (42, 56). In contrast to *M. abscessus abscessus* and *M. abscessus bolletii*, truncation of *erm41* in *M. abscessus massiliense* renders the bacteria susceptible to macrolides (57). The *erm41* truncation is currently used to rapidly predict the outcomes of macrolide therapy in clinical isolates of *M. abscessus* (58). In addition to enzymes that chemically modify antibiotic targets, *M. abscessus* also encodes several proteins that physically bind to drug targets and detoxify them or protect them from subsequent drug binding. A variety of target protection proteins that confer resistance to antibiotics that target the ribosome and RNA polymerase have been described in *M. abscessus*. MAB_2355c and MAB_1846 encode the ARE-ABCF family of target protection proteins that bind to the E-site of the ribosome, displace the bound antibiotic, and confer resistance to macrolides and lincosamides, respectively (59, 60). Additionally, the universally conserved HflX protein (MAB_3042c) dissociates ribosomes stalled in the presence of macrolide and lincosamide antibiotics and sequesters the 50S subunit, thereby making it unavailable for reassembly of the ribosome (61, 62). More recently, target protection was also demonstrated in RNA polymerase wherein MAB_3189 (HelR) confers rifamycin resistance by rescuing and detoxifying rifampicin-bound RNA polymerase (50). Target protection mechanisms, however, confer lower levels of resistance as compared to both enzymatic modification of the drugs and their targets, but nonetheless enable the adaptation of bacteria to the presence of antibiotics and contribute cumulatively to the overall drug susceptibility of *M. abscessus*.

#### Antibiotic efflux

Treatment of *M. abscessus* with efflux pump inhibitors such as verapamil increases its susceptibility to antimicrobials, suggesting that drug efflux contributes to intrinsic antibiotic tolerance in *M. abscessus* (63). The *M. abscessus* genome encodes homologs to efflux pumps belonging to the MFS, ABC SMR, and RND superfamilies, several of which are strongly upregulated in *M. abscessus* upon drug exposure (64). Nonetheless, very few efflux pumps have been shown to be unequivocally involved in *M. abscessus* drug resistance. Furthermore, of the 123 annotated ABC transporters in the *M. abscessus* genome, 62 are unlikely to be transporters as they lack the transmembrane (TMM) domain. Indeed, MAB_2355c and MAB_1846, which contain the signature twin ABC motifs but lack the TMM region, have been shown to belong to the ARE-ABCF family and function in ribosome protection. *Bona fide* efflux pumps in *M. abscessus* thus far are restricted to MmpS-MmpL genes encoded by *MAB_2300-2301* and *MAB_1135c-1134c* that have been described in the efflux of clofazimine and bedaquiline resistance in *M. abscessus* (65) and *MAB_2780c* that confers high-level resistance to spectinomycin (SPC) (66). Curiously, although *MAB_2780c* is ~ 20% and 40% homologous to *M. tuberculosis tap* and *M. smegmatis tetV* genes involved in tetracycline and low-level SPC resistance, the susceptibility of Δ*MAB_2780c* to SPC increases >100 fold, but the susceptibility to tetracyclines remains completely unchanged when compared to the parent wild-type strain (66). The distinction between MAB_2780c and Tap or TetV could possibly be due to key mutations that have altered the number of protein-ligand interactions favoring SPC binding and concomitantly disfavoring tetracycline binding.

#### Adaptive antibiotic resistance

Although some of the antibiotic resistance mechanisms described above are constitutive, several genes responsible for drug resistance in *M. abscessus* are antibiotic-inducible. Central to this adaptive response is WhiB7, a transcriptional activator of the WhiB-like (Wbl) family of transcriptional regulators found exclusively in actinomycetes, which is responsible for the upregulation of several genes including *erm41, MAB_1846*, *MAB_2355c*, *MAB_2780c*, *MAB_3543*, *MAB_4532c*, and *MAB_4395* associated with resistance to macrolides, lincosamides, ketolides, oxazolidinones, various aminoglycosides, and tigecycline (67). Additionally, resistance to bedaquiline, rifamycins, and tetracyclines is also known to be drug-inducible, where the resistance genes are turned on in response to the antibiotics they confer resistance to. The MmpL-MmpS genes involved in bedaquiline resistance and MAB_TetX required for tetracycline resistance are regulated by the TetR family of transcriptional repressors, specifically MAB_2299c and MAB_1497, respectively (53, 65). The expression of MAB_0591 and MAB_3189c—the two major determinants of rifamycin resistance—is also drug-inducible, but by a mechanism that is yet to be known (50).

Remarkably, the majority of the above-described effector genes for antibiotic resistance in *M. abscessus* are not encoded by *M. tuberculosis* or *M. avium* complex (Table 1): two most significant mycobacterial pathogens. This perhaps explains why antibiotics targeting these pathogens are ineffective against *M. abscessus*.

**TABLE 1:** List of genes known to confer antibiotic resistance in *M. abscessus*

Locus tag (MAB_)	Gene	Resistance to	Ortholog in Mtb	Ortholog in MAC	% of clinical Mabs harboring the gene^*a*^	Ref
0591	Adp-ribosyl transferase	Rifamycin	No	No	100	(50, 51)
1135c–1134c	MmpL/S	Bedaquiline	Yes	Yes	98.9	(65)
1496c	Mab_TetX	Tetracycline	No	No	98.96	(53)
1497c	TetR-family regulator	Tetracycline	No	No	100	(53)
1846	Putative ABC transporter	Lincosamide	No	No	100	(59, 60)
2299c	TetR-family regulator	Bedaquiline	Yes	Yes	100	(65)
2300-01	MmpS/L	Bedaquiline	Yes	Yes	100	(65)
2355c	Putative ABC transporter	Macrolide	No	No	100	(59, 60)
2385	streptomycin phosphotransferase	Streptomycin	No	No	98.96	(49)
2780c	Transporter (TetV)	SPC	No	No	100	(66).
2875	Ambler class A β-lactamase	B-lactam	No	No	100	(54)
3042c	HflX	Macrolide/lincosamide	Yes	Yes	100	(61, 62)
3189c	HelR	Rifamycin	No	No	100	(50).
4395	Aminoglycoside 2′-N-acetyltransferase	Aminoglycoside	No	No	100	(48, 49)
4532c	Eis2	Kanamycin	No	No	100	(48)

^*a*^ Limited to 96 isolates, fully annotated reference genomes of which are publicly available at https://www.ncbi.nlm.nih.gov/datasets/genome/?taxon=36809.

### Biofilms and environmental persistence

*M. abscessus* primarily dwells in the soil and aquatic environments, including engineered water distribution systems, in which they likely persist as biofilms (68–71). Clinical evidence further supports the formation of *M. abscessus* biofilms within the thickened alveolar walls, airways, and lung cavity during pulmonary infections in patients with COPD or CF (72–74). Biofilms are homo- or hetero-species multicellular communities of microbes organized on biotic or abiotic surfaces and encapsulated by the extracellular matrix (75). The self-formed gradients of nutrient and oxygen in biofilms give rise to phenotypic heterogeneity in constituent cells, fostering enhanced persistence under antibiotic exposure and other environmental threats (75). Under laboratory conditions, *M. abscessus* spontaneously adheres to the substratum and itself to form biofilms with high levels of drug tolerance, as observed in biofilms of other mycobacterial species (70, 71, 76, 77). Moreover, similar to *in vitro* biofilms of *M. smegmatis* and *M. tuberculosis,* biofilms of *M. abscessus* ATCC 19977 in Sauton’s medium accumulate extracellular free mycolic acids (78–80). In addition, *M. abscessus* biofilms are abundant in extracellular DNA, protein, and carbohydrates (78). Remarkably, biofilms of another clinical *M. abscessus* strain, NJH12, cultured in a synthetic CF medium (SCFM) mimicking the bronchioalveolar lavage composition of CF sputum, presented multiple distinctions from the biofilms of *M. abscessus* ATCC19977 strain in Sauton’s medium (77, 78). For example, unlike the ATCC19977 biofilms in Sauton’s medium, NJH12 biofilms in the SCFM induced a hypoxic response (77, 78), highlighting that differences in strains and/or growth conditions can significantly impact the phenotypes. This is further supported by studies from De et al. and Lian et al. showing that host-adapted mutations in clinical isolates of *M. abscessus* leading to modified lipoarabinomannans altered their ability to form biofilms relative to the *M. abscessus* ATCC19977 strain (81, 82).

### Colony morphotypes and virulence

The ability to cause disease in humans is another unique aspect of *M. abscessus* commonly absent in other rapidly growing mycobacteria. Key features of *M. abscessus* virulence rely on its capacity to modulate its cell surface properties and morphology, which are conditioned by the production of complex glycopeptidolipids (GPL) (83, 84). Whereas the presence of surface-associated GPL confers a smooth (S) morphotype, dysfunctional synthesis or transport of these lipids leads to colonies with a rough (R) texture and irregular margins. This R morphotype endows the bacilli with pronounced aggregative properties and with more virulent and pro-inflammatory phenotypes. In contrast to the S variant, the R form has the propensity to form “cords” on solid or liquid medium and also in macrophages and zebrafish larvae (85–87). Characteristics of the R colony morphotype likely contribute to its enhanced virulence as compared to the S colony morphotype. By proliferating *via* cord formation, which is too large to be efficiently internalized by macrophages, R isolates can evade phagocytosis (86). However, when phagocytosed, R isolates persist intracellularly by inducing the type I interferon-mediated escape into the cytosol and IL-1β production, enhancing bacterial survival in macrophages (88). In addition, R replication within macrophages amplifies the host inflammatory response, exacerbating the pathology and contributing to tissue damage (89, 90). Several reports highlighted the prominence of the R morphotype in patients with severe pulmonary diseases (91, 92). The major molecular mechanism responsible for the S-to-R transition usually includes small insertions/deletions or single-nucleotide polymorphisms within the *gpl* cluster, particularly in *mmpL4b* (encoding a GPL transporter) and in *mps1* or *mps2* (encoding non-ribosomal peptide synthases required for the synthesis of tripeptide-aminoalcohol moiety of GPL) in many isolates (84, 93, 94). Additional studies showed that, in the *M. abscessus* S morphotype, inactivation of *gtf1* or *gtf2* encoding the glycosyltransferases attaching the 6-deoxytalose and first rhamnose to the GPL peptide backbone, respectively, resulted in the S-to-R switch (95). Similarly, deletion of the gene coding for the talose epimerase Tle, converting dTDP-Rha into dTDP-6-deoxytalose (the substrate of Gtf1), leads also to a R morphotype (96). Although the phenotypes of the *gtf1*, *gtf2,* and *tle* mutants were associated with cording, increased abscess formation, and rapid and lethal infection in zebrafish embryos (95, 96), the prevalence of mutations in these genes in clinical isolates remains to be investigated. Recent work identified a small RNA, called B11, as a pleiotropic regulator of multiple pathways playing pivotal roles in the physiology and pathogenesis of *M. abscessus* (90). Deletion of B11 was associated with an R morphotype as a consequence of reduced GPL synthesis, hyper-inflammation, and increased virulence. The mechanistic basis for this regulation is direct binding of B11 to target mRNA *via* specific base pairing. The presence of hypomorphic B11 mutations in clinical strains is consistent with the idea that lower B11 activity may represent an advantage for *M. abscessus* in some clinical situations (90).

While MmpL4 participates in translocation of GPL to the cell surface (97), other MmpL membrane proteins transport various virulence lipids in *M. abscessus* (98). For instance, deletion of *mmpL8_MAB_* correlated with reduced adhesion of *M. abscessus* to macrophages, and attenuation of this mutant correlated with increased zebrafish survival and reduced granuloma formation (99). Lipid analyses indicated that MmpL8_MAB_ is needed for the expression of a glycosyl diacylated nonadecyl diol, which may play a unique role in *M. abscessus* infection (99). Another MmpL member known as MmpL10 transports trehalose polyphleates (100), which are multi-acylated trehaloses contributing to the pathogenicity of *M. abscessus* in macrophages and zebrafish larvae and required for infection by the therapeutic phages BPs and Muddy (101). Other trehalose-based lipids, including trehalose monomycolates, are transported through the plasma membrane by MmpL3, which is a validated drug target for *M. abscessus* drug developments (24, 102). As mentioned earlier, some MmpL proteins (together with MmpS proteins) were reported to confer antibiotic resistance in *M. abscessus* by functioning as efflux pumps (65, 103).

In addition to these canonical R/S morphotypes initially established in the reference strains, clinical isolates can often display intermediates along a spectrum between S and R (104), raising a possibility that S-to-R transition is quantitatively controlled by a regulator of GPL biosynthesis. Indeed, a novel transcription factor, GplR1, appears to regulate the synthesis of GPL (105), although the extent of GplR1 contributions to variations in colony morphotypes among clinical strains remains unknown. A continuous genomic evolution and epigenetic reprogramming under the strong selection pressure of the host immune response, exposure to antibiotics, and other stresses could play a significant role in the development of various colony morphotypes.

### Open pan-genome

**Fig 1:**
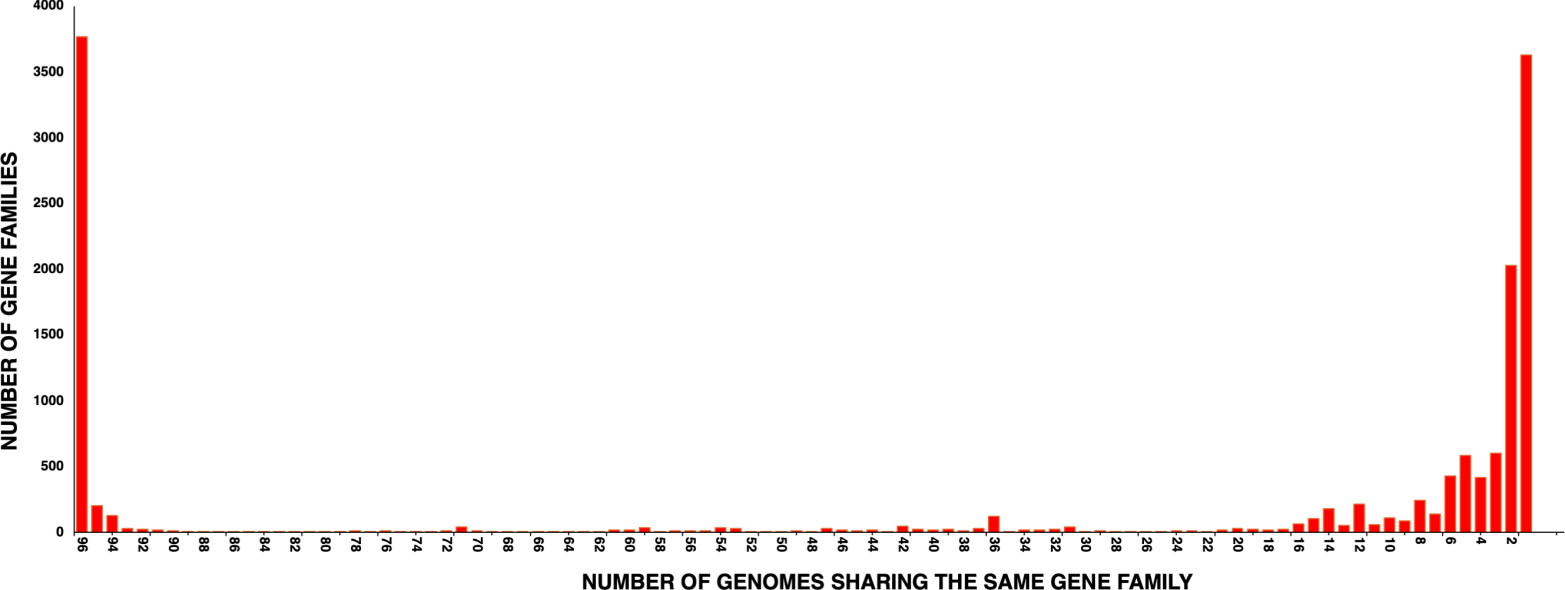
Pan-genome analysis of 96 clinical isolates of *M. abscessus* using Panaroo with parameters: panaroo -i *.gff -o results --clean-mode strict --remove-invalid-genes. The histogram represents the number of gene families shared among the 96 genomes. A total of 3,769 genes were present in all the 96 genomes, thus representing the core genome, whereas 3,627 genes were present just in a single genome. Genes shared by some but not all of the genomes are annotated as accessory genes, whereas those universally shared are core genes.

The pitfalls of limiting the basic research on *M. abscessus* to the type strain ATCC19977 became apparent from the pan-genome analysis that revealed an extensive open, non-conservative pan-genome across 40 isolates, largely driven by HGT (28). At the time of writing this review, completely assembled and annotated genomes of 96 *M*. *abscessus* isolates are publicly available at https://www.ncbi.nlm.nih.gov/datasets/genome/?taxon=36809. A pan-genome analysis of these 96 genomes identified 14,169 genes, of which only 3,769 genes (26.6%) were present in all 96 genomes, therefore called core genomes, while a similar number (3,627 genes, 25.6%) remained confined to just one isolate (Fig. 1). We note that the number of universally conserved genes (3,769) is ~75% of the total genes in a typical *M. abscessus* genome, which is in agreement with the published study (33). A complex evolutionary trajectory of *M. abscessus*, combined with extensive genomic and phenotypic variations among clinical isolates, makes it challenging to develop an experimental framework with a representative strain of the pathogen. Few considerations in experimental designs as described later in this review may help address these challenges.

## FUTURE PRIORITIES IN *M. ABSCESSUS* INVESTIGATIONS

### Looking beyond the type strain

Molecular insights into the unique phenotypes of *M. abscessus*, including extreme drug resistance, have mostly been obtained through basic genetic and biochemical approaches using ATCC19977. However, the genomic diversity across *M. abscessus* clinical isolates limits the scope of ATCC19977 findings. For example, while the key drug resistance regulator in *M. abscessus*, WhiB7, is conserved in all of the 96 annotated genomes of clinical isolates (https://www.ncbi.nlm.nih.gov/datasets/genome/?taxon=36809) and therefore can be reasonably argued as a therapeutic target, MabTetX is present in 95 of the isolates, implying that the strategy of targeting this gene will exclude ~1% of the clinical isolates. Moreover, fundamental questions also emerge as to whether these isolates lacking MabTetX are more sensitive to tetracycline or whether they deploy an alternative resistance mechanism. Any future genetic studies on ATCC19977 or its isogenic R variant are expected to encounter these questions, and appropriate steps should be incorporated in the experimental design for an informed and inclusive outcome. For this purpose, we have developed a web-based tool to determine the extent to which a gene of interest is conserved across the 96 aforementioned clinical isolates (https://abscessus.wadsworth.org/). The pan-genome analysis using this tool can be performed in four sequential steps: i) “Select Strain,” ii) Click on “current” or “old locus tag names,” iii) select gene from the drop-down menu, and iv) Click “Query Gene.” The output provides a list of all the genomes containing the gene. This information can serve as a reasonable starting point in the experimental design. While universally conserved genes like *whiB7* (106), or the ribosome hibernation factor *mpy* (107), forming the core genome will remain high-priority therapeutic targets, accessory genes would require a careful assessment of their value in translational research given the limited scope of their function. The accessory genes also open up new possibilities of exploring functional substitutes in strains lacking these genes. Further expansion of this web-based tool with a greater number of genome data sets of *M. abscessus*, *M. bolletii*, and *M. massiliense* will offer a high-resolution analysis of the status of a gene of interest.

### Optimizing *in vitro* growth conditions

In a recent study, Akusobi et al. experimentally demonstrated that the set of essential genes required for carbon metabolism *in vitro* varied across *M. abscessus* isolates (26), implying that a given growth condition can potentially have a strain-specific effect on the doubling time, gene expression, and drug sensitivity. Thus, a consideration of growth conditions for *in vitro* investigation of *M. abscessus* becomes an important component of experimental design, and the choices will depend on the strains being tested as well as questions being addressed. While the two originally used *M. abscessus* growth media, 7H9OADC-Tween80 and Sauton’s medium, have been adopted from *M. tuberculosis* and *M. smegmatis* studies, more recent studies also include SCFM and artificial CF sputum**,** which closely represent the chemical composition of lung airways and seem to profoundly alter the global gene expression pattern, the surface lipid profile, and drug sensitivity of *M. abscessus in vitro* (77, 108, 109). It is noteworthy that human sputa expectorated either from tuberculosis (110) or CF patients (111) are sufficiently zinc-deprived to induce crucial zinc starvation response in *M. tuberculosis* or *Pseudomonas aeruginosa,* respectively. Zinc starvation in *M. smegmatis* and *M. tuberculosis* induces stepwise changes in the ribosomes (107, 112–114). First, the ribosome is remodeled through replacement of the ribosomal proteins containing the zinc-binding CXXC motif with their motif-free paralogs (107, 112). Upon further zinc depletion to a growth-restrictive concentration, the remodeled ribosome is targeted for hibernation by mycobacterial protein Y (Mpy) (107, 112). Importantly, both remodeling and hibernation of ribosomes induce antibiotic resistance in mycobacteria (107, 112, 115), implying that the low-zinc environment of the human lung airways likely contributes to increased antibiotic resistance in resident mycobacteria. Our unpublished work shows that *M. abscessus* similarly responds to zinc starvation by remodeling and hibernating the ribosome (Treen et al. manuscript under preparation). Thus, we propose that depleting zinc from the sputum-mimicking medium will further simulate the lung environment, thereby providing new insights into the zinc-responsive physiological changes in *M. abscessus* with an impact on antibiotic resistance. In summary, a strategic experimental approach for *in vitro* characterization of an *M. abscessus* gene should consider its universality and its requirement in a growth condition that most closely resembles the lung airways.

### Host-adapted pathogenomic evolution in animal models

The genomic diversity among clinical isolates of *M. abscessus* raises key questions about the types of evolutionary path followed by each strain during its transition from an environmental lifestyle to a host niche, and how host-induced selection pressure dictates such evolution. Addressing these questions may lead to an understanding of why some strains, called dominant circulating clones (DCCs) (116), are clinically more prevalent than others. However, two key bottlenecks exist in approaching these questions. First, relatively little is known about the genetic and phenotypic characteristics of environmental *M. abscessus* strains (117, 118). Second, suitable animal models that can inform us about both the mechanisms of colonization as well as the chronic persistence of *M. abscessus* still remain to be optimized.

#### Relationship between environmental and clinical isolates of *M. abscessus*

Thomson and colleagues pioneered the investigation of relatedness between environmental and clinical isolates of *M. abscessus* using samples from Brisbane, Australia (118). Interestingly, some clinical isolates were indistinguishable from environmental isolates with respect to their repetitive genome sequences, thereby suggesting environmental reservoirs as possible sources for human infections (118). Their study also discovered that using solid media during recovery increased the detection frequency, perhaps explaining why the use of liquid medium in prior methods had a poor success rate (118). Honda and colleagues have developed a sizable collection of environmental isolates of *M. abscessus* and related clinical isolates from Hawaii, USA (119)*,* and appear to have begun investigating their relatedness using the whole-genome level (120). However, these cross-sectional studies may offer limited understanding of the adaptive changes in *M. abscessus* necessary for its survival in hosts. Perhaps a systematic approach would involve longitudinal analysis of the genome sequences of an environmental isolate upon infection in an animal model. A high-resolution mapping of genomic variations throughout the duration of infection can potentially identify mutations that facilitate *M. abscessus* adaptation in hosts. However, animal models recapitulating early and chronic stages of *M. abscessus* infection would be necessary to address this gap. Findings from such studies will complement those reported by Bryant et al. that mapped the evolutionary trajectory of DCCs (116).

#### Zebrafish model of *M. abscessus* infection

The current understanding of the chronological events during early stages of *M. abscessus* infection is largely afforded by non-invasive and real-time imaging of the zebrafish infection model (86, 121). This model revealed the distinctive pathophysiological characteristics and infection outcomes of the R or S variants. Original work in the zebrafish embryo, possessing only innate immunity, identified the typical extracellular R cords, which can resist internalization by macrophages and neutrophils due to their excessive size, emphasizing a mechanism of immune subversion. In addition, cords can induce formation of abscesses, which contribute to pathogenesis and tissue damage, leading to acute and lethal infection (86). Indispensability of macrophages in controlling *M. abscessus* infections has been established by infecting macrophage-depleted zebrafish embryos with the R variant of *M. abscessus* that led to pronounced bacterial burst, enhanced cording, and larval killing (86). Subsequent loss-of-function studies combined with fluorescent reporter zebrafish lines and high-resolution imaging unraveled the contribution of TNF signaling and IL-8-mediated neutrophil recruitment toward protective granuloma-forming immunity against *M. abscessus* (122). These findings were consistent with the previously obtained results in the mouse model (123)**,** affirming the suitability of the zebrafish model to investigate *M. abscessus* pathogenesis and pathogenomic evolution. Zebrafish larvae were also used to evaluate the virulence of genetically defined *M. abscessus* mutants (121), targeting various genes or pathways, such as the *gpl* locus (95, 96), MmpL lipid transporters (97, 99), or the type VII secretion system (124). The embryo model has also been very useful to address the possible connection between dysfunctional cystic fibrosis transmembrane conductance regulator (CFTR) and vulnerability to *M. abscessus* disease observed in CF patients. A CFTR-depleted zebrafish model, which recapitulates CF immunopathogenesis, established a correlation between the loss of CFTR function and increased susceptibility to *M. abscessus* infection (125). The use of fluorescent-labeled strains has also been exploited in the adult zebrafish model, which possesses functional innate and adaptive immunity, for evaluating chronic infection with both S and R morphotypes of *M. abscessus* (126). In adult fish, *M. abscessus* S morphotype progressively replicates through a 28-day infection period, while the R variant maintains a stable bacterial burden but with a greater number of necrotic granulomas relative to the S variant (126).

Most of these above-mentioned studies were conducted using the type strain ATCC19977 (CIP104536^T^) for which efficient genetic tools were developed. Meanwhile, challenges associated with the use of clinical isolates in the zebrafish model are yet to be fully recognized. For example, while infection studies in the zebrafish model rely on the use of fluorescent reporter strains, many *M. abscessus* clinical isolates are known to be refractory to transformation by exogenous DNA. In addition, extreme antibiotic resistance in some clinical strains, particularly to kanamycin and hygromycin, which are often used for selecting transformants *in vitro*, can prevent their genetic manipulation. This can, however, be circumvented by selection under high concentrations of antibiotics (up to 1 mg/mL hygromycin) and usage of fluorescent protein markers expressed from the recombinant constructs (100, 127). Despite these limitations, infection of zebrafish with clinical strains followed by WGS of pre- and post-infection population can potentially identify mutational hotspots in the chromosome associated with host-adapted evolution during early stages of infection. However, due to a relatively short lifespan of zebrafish larvae, this model is not ideal for investigating *M. abscessus* persistence during chronic infection, for which higher animal models like mouse models could perhaps be useful.

#### Mouse model for chronic *M. abscessus* infection

Unlike *M. tuberculosis*, *M. abscessus* has low virulence in mouse models, and establishing a persistent (>10 days) pulmonary *M. abscessus* infection in mice remains a challenge. A recent review on chronic *M. abscessus* models highlights variability in the infection course, bacterial growth, pathology, severity, mortality, and antibiotic response (128). One recent study examined 15 mouse strains with various immunological, lysosomal, and connective tissue disorders, along with their respective controls (129). A total of 11 of these 15 mouse strains, including immunodeficient mice, cleared *M. abscessus* within 28 days of infection. Importantly, this resistant group included CFTR KO mice (CF model), although CFTR KO mice do not develop the progressive CF-like lung pathology seen in humans. Similarly, NOS-2 KO, Marfan, Hurler, and NPC mice exhibited infection courses comparable to control immunocompetent mice such as BALB/c and C57BL/6. In four mouse strains—two new (NSG and NRG) and two previously reported strains, GM-CSF KO and IV-infected SCID-Beige mice—the chronic infection lasted between 56 and 90 days. Three of these strains—SCID Beige, NRG, and NSG—are particularly immunocompromised, lacking T, B, and NK cells. The NRG and NSG strains also lack the IL-2R gamma chain. While SCID Beige and NSG/NRG strains may help test anti-*M*. *abscessus* drugs, these models do not fully reflect human disease caused by *M. abscessus* infection as patients typically do not experience such extensive systemic immunodeficiency. Additionally, lung pathology in these mouse strains was minimal during chronic *M. abscessus* infection. Moreover, GM-CSF deficiency affects macrophage maturation and antimicrobial function. Mice lacking GM-CSF (GM-CSF KO) show a distinct acute infection phase with logarithmic bacterial growth, making it an ideal strain for testing compounds that are most efficient over a short period against actively replicating bacilli (130, 131), as well as a chronic infection state lasting at least 56 days. However, bacterial burdens during the chronic phase display higher variability. Additional models of chronic infection utilize low-dose dexamethasone to create an immunosuppressive state that limits antimicrobial activities elicited by the immune responses. Dexamethasone dosing is required daily; skipping doses reduces bacterial lung burden (132, 133). A recent study replaced dexamethasone with once-weekly immunosuppression using cyclophosphamide administration to induce immunosuppression and facilitate chronic *M. abscessus* infection (134). Instillation of agar bead-embedded *M. abscessus* in mice is also used to obtain chronic infection that can last up to 45 days after inoculation and can form granuloma-like lesions in immunocompetent mice (135–137). Local lung alterations caused by beads presumably facilitate colonization by bacteria. Taken together, these recent advances in the development of a chronic infection model of *M. abscessus* will potentially facilitate experiments toward a deeper understanding of the host-adapted pathogenomic evolution in the pathogen.

### Novel therapeutic approaches

The inefficacies of anti-tuberculosis drugs against *M. abscessus* have propelled the efforts in developing novel therapeutic approaches that include using new combinations of existing drugs (138–140) or developing new agents, some of which emerged from the molecular insights into the resistance mechanism (131, 141). For example, based on the discovery of Hurst-Hess et al. that TetV efflux pump minimizes the intracellular concentration of SPC in *M. abscessus* (66), Phelps et al. developed a new N-ethylene-linked SPC derivative that remains resistant to efflux by TetV, thereby avoiding this resistance mechanism to efficiently kill *M. abscessus* (141). Similarly, Dartois et al. developed a novel derivative of rifamycin that efficiently targets *M. abscessus* by overcoming intrinsic resistance mediated by ADP-ribosylation and minimizing the induction of cytochrome P450 3A4, which rapidly metabolizes the drug (131). However, clinical outcomes of these approaches in treating human infections remain to be assessed.

Perhaps the most surprising and remarkable clinical outcome in the treatment of human *M. abscessus* infections has emerged through compassionate use of mycobacteriophages that produced clinically favorable outcomes in over 50% cases (142–144), underscoring phage therapy as a viable adjunct to antibiotics, although significant challenges currently limit this approach from broader implementation. First, the narrow host range preference of each phage isolate necessitates pre-screening of a patient-derived *M. abscessus* strain against a collection of mycobacteriophages. Second, the S-colony variants of *M. abscessus* are generally recalcitrant to all mycobacteriophages tested so far, whereas the R-colony variants exhibit varying degrees of phage susceptibility (93). Third, intravenous injection of a high concentration of phages for several months during therapy increases the chance of introducing potentially harmful genetic elements such as toxin-encoding genes in the host (145). Lastly, the emergence of phage-resistant *M. abscessus* mutants during treatment remains a distinct possibility (145). Some of these challenges, however, can be mitigated by using purified and well-characterized phage components responsible for *M. abscessus* killing. One such component is lysin B protein (LysB), which is a member of the serine hydrolase protein superfamily that is unique to mycobacteriophages and primarily hydrolyzes the ester bonds linking mycolic acids with a sugar moiety in mycolylarabinomannan-peptidoglycan complex or trehalose dimycolate (TDM) of the cell envelope (146). Based on a previous discovery of mycobacterial lysis by recombinant hydrolase of TDM (147), Yang et al. demonstrated that recombinant LysB from mycobacteriphage D29 can rapidly and efficiently neutralize multiple *M. abscessus* clinical isolates *in vitro* and in an acute mouse infection model (148). Their finding was later corroborated by others who reported killing of intracellular *M. abscessus* by a cocktail of lytic enzymes including LysB (149). Importantly, S-colony morphotypes were equally susceptible to LysB (148), making this protein a powerful alternative to phages in targeting these variants. Thus, natural and engineered LysB proteins can be potent therapeutic adjuncts, which, when carefully combined with antibiotics, can potentially shorten the treatment regimen for *M. abscessus* infections. Lastly, host-directed therapy—an approach that modulates the host immune response to infections—is also emerging as a potential strategy against NTM diseases (150).

## CONCLUSION

**Fig 2:**
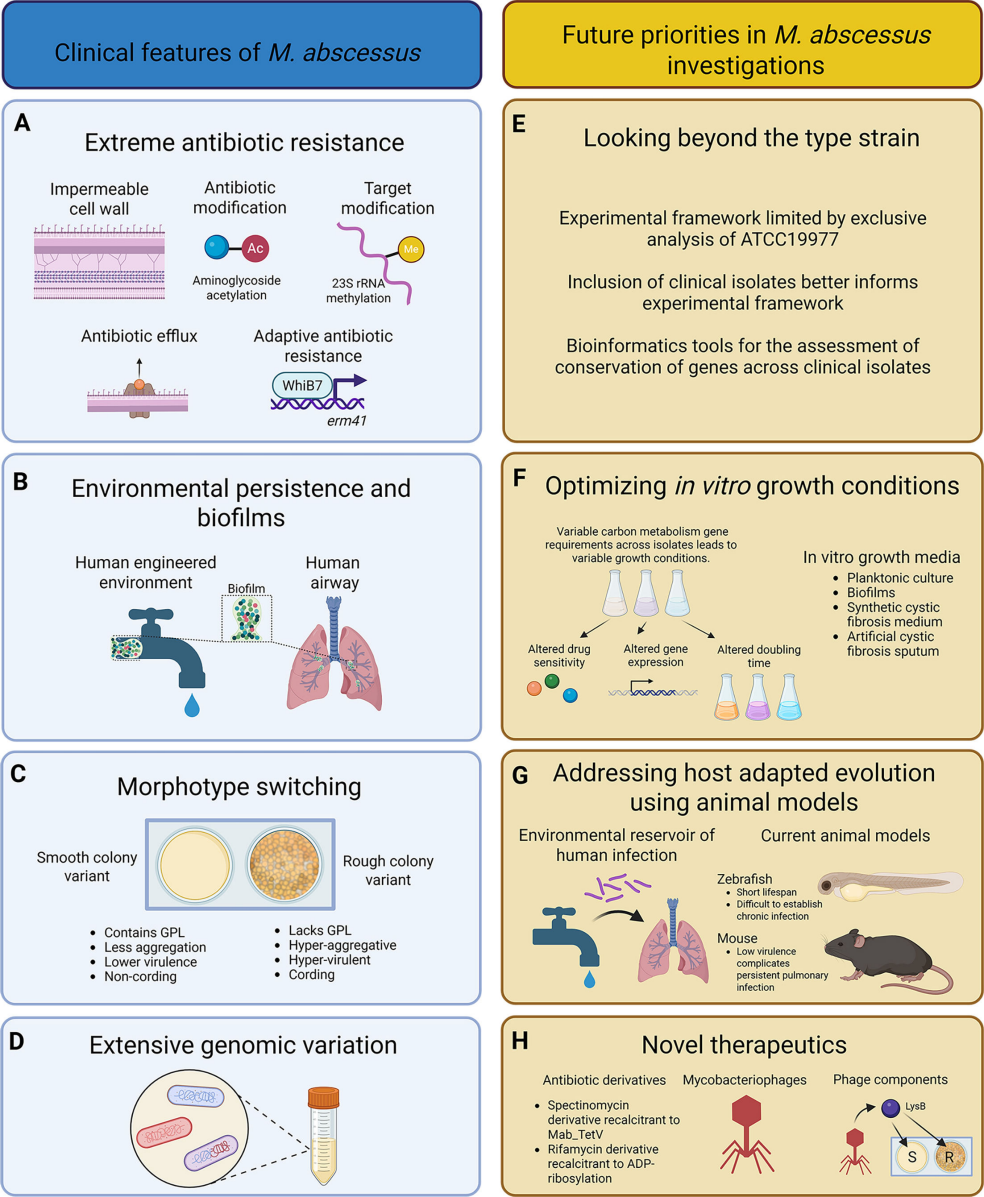
A graphical abstract highlighting the key features of *M. abscessus* (**A–D**) and future research priorities (**E–H**).

A rising trend in *M. abscessus* infections in people with underlying conditions and their recalcitrance to antibiotics demands urgency in the development of short and effective treatment strategies, which can be best accomplished through studies that unravel the biological implications of genomic variations among the clinical isolates. In highlighting some common challenges encountered in *M. abscessus* studies and outlining possible considerations, this review presents a roadmap for future *M. abscessus* research in key topics of high significance (see Fig. 2), advancing which could potentially yield new therapeutic strategies.
